# Internet- and App-Based Stress Intervention for Distance-Learning Students With Depressive Symptoms: Protocol of a Randomized Controlled Trial

**DOI:** 10.3389/fpsyt.2019.00361

**Published:** 2019-05-21

**Authors:** Mathias Harrer, Jennifer Apolinário-Hagen, Lara Fritsche, Marie Drüge, Ludwig Krings, Korinna Beck, Christel Salewski, Anna-Carlotta Zarski, Dirk Lehr, Harald Baumeister, David Daniel Ebert

**Affiliations:** ^1^Clinical Psychology and Psychotherapy, Institute for Psychology, Friedrich-Alexander-University Erlangen-Nuremberg, Erlangen, Germany; ^2^Department of Health Psychology, Institute for Psychology, University of Hagen, Hagen, Germany; ^3^Division of Online Health Trainings, Innovation Incubator, Leuphana University, Lüneburg, Germany; ^4^Clinical Psychology and Psychotherapy, University of Ulm, Ulm, Germany

**Keywords:** distance-learning students, randomized controlled trial, stress management, depression, telemedicine, Internet, mobile app

## Abstract

**Background:** Mental disorders are highly prevalent among university students. Distance-learning students are particularly burdened and have limited access to conventional university health services. Interventions for stress are sought after in distance learners and may help increase treatment coverage. Internet-based interventions have been shown to be effective in preventing and treating depression, but it remains unclear if interventions directed at academic stress also have this potential.

**Aim:** The trial presented here investigates the effectiveness of an Internet- and App-based stress intervention in distance-learning students with elevated levels of depression.

**Methods:** A sample of *N* = 200 students of a large German distance university with elevated levels of depression [Center for Epidemiological Studies’ Depression Scale (CES-D) ≥ 16] will be randomly assigned to either an Internet- and App-based stress management intervention group (IG) or a control group (CG) receiving an Internet-based psychoeducational program for academic stress. The IG consists of eight Internet-based sessions promoting stress management skills using cognitive–behavioral and problem-solving techniques. A mobile App will be employed to facilitate training transfer. Self-report data will be assessed at baseline (T0), post-treatment (T1; 7 weeks), and 3-month follow-up (T2). Potential moderators will be assessed at baseline. The primary outcome is depression (CES-D) post-treatment. Secondary outcomes include mental health outcomes, modifiable risk and protective factors, and academic outcomes. Data will be analyzed on an intention-to-treat principle along with sensitivity analyses to assess the robustness of findings. Additional health economic analyses will be conducted.

**Discussion:** Results will provide the basis to assess the acceptance and effectiveness of Internet-delivered stress interventions in distance-learning students with symptoms of depression.

**Ethics and dissemination:** The study has been reviewed and approved by the University of Erlangen-Nuremberg ethics committee (Erlangen, Germany; 33_17 Bc). Results of the study will be disseminated through peer-reviewed publications.

**Trial Registration:** German Clinical Trial Registration (DRKS), identifier DRKS00011800

## Background

Previous research documents that mental disorders are highly prevalent in tertiary education students (1–4). Depressive disorders are particularly common, with 12-month prevalence estimates ranging from 4.5% to 18.5% (1, 2, 4). Mental illness in the college years is associated with a broad range of adverse personal and societal outcomes, including lower academic performance (5–7) and college retention (8, 9), as well as worse physical health (10), and role functioning (11,12) in later adulthood. Suicidality is strongly associated with mental health disorders (13) and highly prevalent among university students (14–16), making it the second largest cause of mortality in this population (17).

Compared to the general university student population, distance learners may be particularly at risk for suffering from a mental disorder. Distance-learning services are frequently used by individuals over 30, by employees attaining qualifications in addition to holding a job, or by parents (18, 19). This may result in a more complicated educational environment for affected individuals (19, 20). Results from a survey among 5,721 German distance learners indicate that this population, compared to on-site students, faces great strain due to having to meet the demands of multiple societal roles (20). Resulting mental health problems have been associated with worse academic attainment in distance learners (21).

There is evidence that the large majority of students suffering from mental disorders do not receive any treatment for their mental health problems (1). Attitudinal factors such as personal stigma (22) or preference to deal with poor mental health on one’s own (23) have been discussed as major barriers in help-seeking.

Internet-based interventions have gained increasing attention as an instrument to foster health care utilization in adults (24). In recent years, the potential of Internet-based interventions to address mental health issues among tertiary education students has also become increasingly evident (25, 26). Contents can be easily accessed through the Internet, and Internet-delivered programs provide high scalability (27–29). Implementing Internet-based interventions in distance-learning university settings could represent a particularly promising approach, as distance-learning students commonly do not have direct access to on-site student counseling services.

Inclination to use Internet-based services has been found to be greatest among students reporting high levels of perceived stress (30, 31). In a survey among distance-learning students, interventions for relaxation and stress management were the most sought after, with 66.9% and 54.8% indicating interest in participation (20). Previous research suggests that Internet-based interventions for stress are often used by students with clinically relevant levels of depression who did not seek help through traditional healthcare channels (32). Disseminating Internet-based stress interventions could therefore be a non-stigmatizing way to increase treatment coverage among students suffering from depressive symptoms.

It is well established that perceived stress is a common contributor to the development of depression (33) and negatively affects its clinical course (34, 35). Internet-based stress interventions not only have been shown to be effective in reducing perceived stress (27) but also have been found to show moderate to high effects on depressive symptoms [Cohen’s *d* = 0.52–0.95 (32, 36–38)] even in participants with clinically relevant levels of depression at baseline [*d* = 0.67–1.19 (32, 39)]. These effects are comparable to the efficacy of conventional types of psychotherapy for depression [*d* = 0.62–0.92 (40)]. Together, this suggests that Internet-based stress interventions could be an effective way to reduce depression. Internet-based interventions for depressive symptoms have proven their effectiveness in preventing (41, 42) and treating (43) depression. However, no prospective trial has yet examined if Internet-based interventions for academic stress also have this potential in students with elevated levels of depression. It is also largely unknown if such interventions can have an impact on students’ academic productivity and work impairment. In general, it is still unclear which characteristics explain the heterogeneity of effects in the prevention and treatment of depression (44, 45). A broad assessment of variables predictive of differential intervention outcomes, risk and protective factors associated with the onset and maintenance of depression, as well as theoretically derived variables within intervention studies has therefore been proposed to increase our understanding of effect modifiers underlying interventions for depression [(44, 46); for an overview of potential effect modifiers in the prevention and treatment of depression, see Refs. (46, 47)].

The goal of the present study is therefore to i) evaluate the effects of an Internet- and App-based stress intervention in distance-learning students with heightened levels of depression on mental health outcomes, modifiable risk and protective factors for mental health disorders, and functioning outcomes when compared to an active control group (CG) receiving an Internet-based psychoeducation program on stress management; ii) to investigate the moderators of potential treatment effects; iii) to assess the help-seeking intentions of study participants; and iv) to assess the interventions’ health economic benefits when implemented into routine care. We hypothesize the Internet- and App-based intervention group (IG) to be more efficacious when compared to the active CG receiving the psychoeducation program.

## Methods

### Study Design

We will conduct a two-armed randomized controlled trial (RCT) comprising two conditions: the IG, receiving an Internet-based intervention with feedback on demand (*StudiCare Fernstudierende*), and a waitlist CG receiving psychoeducation during the study phase, and access to *StudiCare Fernstudierende* after the study. Both study conditions will have full access to treatment as usual (TAU; including general practitioner visits, counseling services, psychotherapeutic and psychiatric treatment or other forms of primary, secondary, or tertiary care). The trial will be conducted and reported in accordance to the Consolidated Standards for Reporting Trials (CONSORT) Statement (48) and the Guidelines for Executing and Reporting Research on Internet Interventions (49). We aim to communicate results of this study through peer-reviewed publications in psychiatric or eHealth journals. In addition, code scripts for all statistical analyses will be made publicly available on an Open Science Framework repository (OSF; www.osf.io).

**Figure 1:**
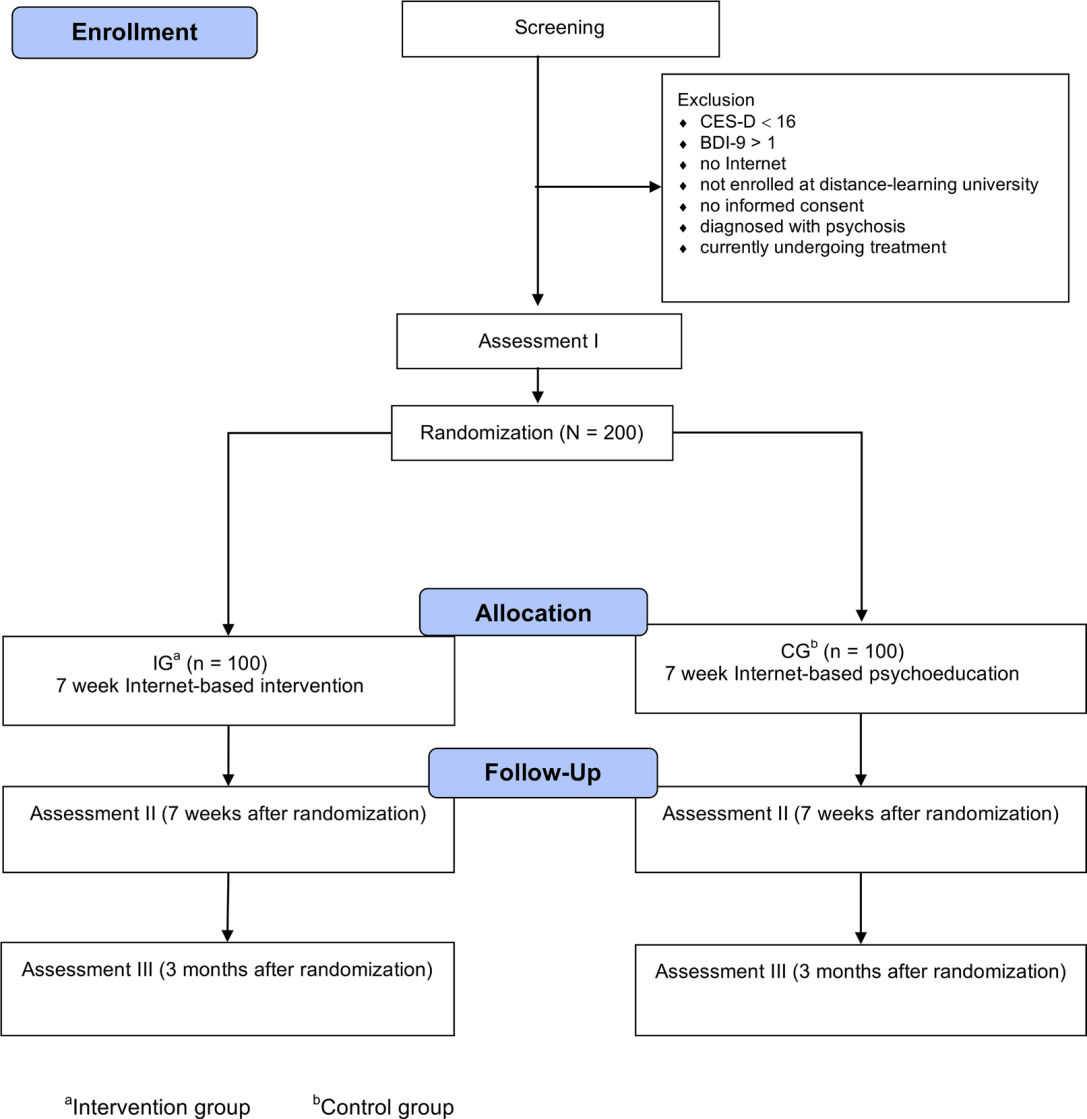
Study flow.

Assessments will take place at baseline (T0), post-treatment (T1; 7 weeks after randomization), and 3-month follow-up (T2; see **Figure 1** for a detailed overview of assessment points). Self-report data are collected using a secure online-based assessment system (Advanced Encryption Standard, 256-bit encryption). All procedures involved in the study are consistent with the generally accepted standards of ethical practice. The study was approved by the University of Erlangen-Nuremberg ethics committee (Erlangen, Germany; 33_17 Bc). The trial is registered in the German clinical trials register (DRKS00011800). The trial proceedings presented here are reported in accordance to the Standard Protocol Items: Recommendations for Intervention Trials (SPIRIT) statement (50). The populated SPIRIT checklist can be found in **Table S1 in the Supplementary Material**. The SPIRIT figure is presented in **Figure S1 in the Supplementary Material**.

### Participants

Students will be included if they i) experience elevated levels of depression measured by a score of ≥16 on the German version of the Center for Epidemiological Studies’ Depression Scale (CES-D) 20-item version [ADS (51), indicating subthreshold to full-blown symptoms of depression during the last 2 weeks], are enrolled in a ii) bachelor’s or iii) master’s degree program at a large German distance-learning tertiary education facility (*FernUniversität in Hagen*) by the beginning of the intervention, iv) are at least 18 years old, v) have Internet access, vi) declare willingness to provide self-report data at all three assessment points, and vii) give informed consent. Exclusion criteria will be i) self-reported dissociative symptoms or psychosis, currently or in the past, or ii) a considerable risk for suicide as indicated by a score higher than 1 on item 9 of the German version of the Beck Depression Inventory [BDI-II (52); “I feel I would be better off dead” or “I would kill myself if I had the chance”]. Students will not receive monetary compensation for participating in the intervention.

### Recruitment

Participants will be recruited in German-speaking countries (Germany, Austria, Switzerland) *via* social media advertisement, university press reports, and information letters distributed through the distance-learning universities’ mailing list. Potential participants will be able to declare interest for partaking in the study by filling out an online registration form on a website created for the intervention that also contains further information about the intervention and eligibility criteria.

### Assessment of Eligibility and Randomization

Individuals who declare their interest in participating in the study will receive an online letter with detailed information about the study procedure and will be asked to provide an e-mail address as well as a first and last name (which may be pseudonyms) for their intervention platform profile. Applicants will be informed that withdrawal from the study is possible at any time without negative consequences and that all collected case data can be deleted on request during the study. Participants will then be asked to fill out the online screening questionnaire.

Individuals fulfilling all inclusion and none of the exclusion criteria will be asked to fill out an informed consent form. Individuals who provide us with their informed consent will be invited to fill out the baseline assessment, which will take approximately 30 min to complete. Subsequently, individuals will be randomly allocated to either the IG or the CG. Randomization will take place at a ratio of 1:1 and a block size of 2 using an automated computer-based random integer generator (*Randlist*, Datinf GmbH, Tübingen, Germany) and will be performed by a researcher who is not involved in the study. Participants will not be blinded to study conditions, yet during the randomization process, the allocation will be concealed from participants, researchers involved in recruitment, and e-coaches.

### Participant Safety

A standardized operating procedure will be followed in case individuals are defined as showing an elevated risk for suicide. During the screening process, individuals will be excluded from the study and given detailed information about emergency contact numbers and treatment options for depression based on the German S3-guidelines for unipolar depression (53). Individuals will also be asked to see their physician or a psychiatrist as soon as possible to initiate psychiatric or psychotherapeutic treatment. The same procedure will be followed if participants who were included in the trial show symptoms of suicidality throughout the study phase (i.e., by mentioning symptoms of suicidality when contacting their designated eCoach, or when responses to intervention content hint at potential suicidal ideation). These participants will be contacted additionally to determine the severity of suicidal symptoms and discuss next treatment steps. This process will be supervised by an experienced clinician at the first author’s host institution.

### Sample Size

We aim to include *N* = 200 participants, allowing for a between-trial arm group comparison against a statistically relevant effect size threshold of *d* = 0.40, a power (1 − β) of 80%, and an alpha of 0.05 (two-tailed) for the intention-to-treat (ITT) analysis (54). A recent meta-analytic review for Internet-based stress interventions reported effect sizes of *d* = 0.64 for perceived stress in guided interventions but considerably smaller effect sizes for unguided programs (*d* = 0.34 for depression, *d* = 0.32 for anxiety) (27). Results for Internet-based interventions addressing psychological distress in tertiary education are mixed, ranging from non-significant findings to moderate-sized effects in favor of the respective intervention (55–59). Thus, we aim for an effect size of *d* = 0.40.

### Intervention Platform

The technology platform used to deliver the intervention in the IG as well as the psychoeducational material in the CG is provided by Minddistrict. This company is responsible for the provision and maintenance of the platform. Its content management system is used to upload interventions, add participant and eCoach profiles, and fill out questionnaires. The platform conforms to all required quality standards and operates according to the ISO 27000 and NEN 7510 norms. All data are securely stored on ISO 27000 certified servers and transmitted using HTTPS with SSL certificates (AES-256 and SHA-1, 2048-bit RSA). Unauthorized access to the platform is not possible.

### Intervention

This trial will evaluate the *StudiCare Fernstudierende* intervention. It was adapted from *StudiCare Stress* (32), an Internet- and App-based intervention for college students. A detailed description of this pilot program can be found elsewhere (32, 60). Together with the preceding pilot intervention, the program is based on *Get.On Stress*, an Internet-based stress intervention for employees (36, 37), which was adapted to a university student context.

In terms of therapeutic content, *StudiCare Fernstudierende* only marginally deviates from *StudiCare Stress*. To tailor the intervention to distance-learning students’ needs, one new student testimonial is introduced and will lead participants through the intervention. The testimonial represents an elder student with children. The testimonial was created to address the specific problems of non-traditional distant-learning students, such as limited time for studying, having to take care of children, or facing financial pressure.

Conceptually, the Internet intervention aligns with Lazarus’ transactional model of stress (61). It adheres to a two-component structure, incorporating problem- and emotion-focused coping through emotion regulation strategies. In problem-focused coping, cognitive behavioral strategies are applied to solve personal problems and to reduce and eliminate stressors (62). Emotion regulation refers to processes through which individuals monitor, evaluate, and modify emotions to reach relevant goals (63).

There are various stressors in university students’ lives that might be potentially solvable by applying problem-solving strategies. Problem-solving heuristics have been shown to be a practical method for addressing stressors and have been proven to be effective in improving mental health outcomes (64, 65).

To tackle situations in which problem-focused strategies are inapplicable, emotion regulation techniques may be employed to control negative emotions associated with unsolvable problems. Current findings indicate that deficits in the ability to detect and regulate negative emotion may contribute to the wellbeing of individuals, as well as the development and trajectory of mental disorders (66–69). Emotion regulation strategies have shown to be an adequate therapeutic instrument in alleviating various symptoms of mental illness (70–72).

The intervention contains seven modules, each of which can be completed in one session (see **Table 1**). Completing one session is estimated to take about 30–90 min, and participants will be advised to work on one or maximum two modules per week. The intervention is therefore intended to last about 5–7 weeks. After modules 2–7, participants will be offered optional add-on mini-modules. These mini-modules cover information and exercises on student-specific topics of interest: social support, rumination and worrying, time management, procrastination, test anxiety, sleep, motivation, nutrition and exercise, dealing with writer’s block, and concentration. An additional booster session allowing participants to recap and rehearse previously learned strategies will be offered 2 weeks after completion of the main modules. Therapeutic content is presented as an illustrative story of a backpacking trip around the world, with each module representing a new continent.

**Table 1:** Modules of the intervention (StudiCare Fernstudierende).

Session	Name	Content	
1	Introduction	Psychoeducation, information about stress and preview of subsequent sessions	
2	Problem-solving	Stress management strategies, systematic problem-solving using a 6-step problem solving heuristic	
3	Muscle and breath relaxation	Information on basic principles of muscle and breath relaxation, audio exercises for daily usage	
4	Mindfulness	Coping with self-criticism, mindfulness exercises	
5	Acceptance and tolerance	Dealing with unsolvable problems, psychoeducation on and exercises for acceptance and tolerance of unpleasant emotions	
6	Self-compassion	Self-criticism in precarious situations, defusion of self-worth and performance, exercises for positive self-support, overcoming dysfunctional perfectionistic thought-action patterns	
7	My master plan	Recognizing physiological warning signs, creating a plan for the future	
8	Booster session	Further information on self-help and psychotherapy, evaluation of training transfer, recap of all sessions, repetition of previous exercises	
2–7	*Elective mini-modules*	*Social support*	communication styles, receiving and providing support
		*Rumination and worrying*	reflection on positive and negative aspects of worry, coping with uncertainty
		*Time management*	effective time scheduling, common planning fallacies, learning to prioritize
		*Procrastination*	identifying situations in which procrastination occurs, strategies to reduce procrastination
		*Test anxiety*	effective studying techniques, using paradoxical intentions, de-catastrophizing blackouts
		*Sleep*	sleep restriction
		*Motivation*	finding reasons for lacking motivation, exercising delay of gratification
		*Nutrition and exercise*	creating an individual eating and exercise schedule, dealing with relapses
		*Dealing with writer’s block*	reasons and mechanisms for writer’s block
		*Concentration*	audio-based concentration exercises

Some technical features were added to strengthen the transfer of intervention content into participants’ everyday life. Homework assignments are given after every module to practice techniques presented during the session. To keep track of mood fluctuations and describe experiences in transferring acquired knowledge, a personal diary App is introduced in the first lection and can be downloaded afterward. After every module, audio files and module summaries can be accessed, containing exercises to be worked on until the next session.

### Psychoeducation

Participants in the active CG will receive psychoeducational information about cognitive, emotional and physical determinants, symptoms, and outcomes of psychosocial stress in general and with respect to distance-learning students. The psychoeducation lessons are delivered through the same platform as the intervention in the IG. In contrast to the intervention, the psychoeducation lessons are largely text-based and do not contain any interactive components. In line with the weekly intervals of the IG, the psychoeducation consists of seven main sessions and one booster session, and is designed to be completed within 5–7 weeks. An overview of the psychoeducation material provided in each module is presented in **Table 2**.

**Table 2:** Psychoeducation modules in the control group.

Session	Name	Content
1	Introduction	Prevalence and types of stress; biological response to stress; effects of stress on emotions, thought, somatic symptoms
2	Causes of stress	Common stressors among students; Lazarus’ transactional model of stress
3	Does stress have the same effect on all individuals?	Short and long-term consequences of stress; inter-individual differences in stress response
4	What effect does stress have on the body?	Physiological response to stressors; evolutionary background of stress reactions; stress and performance
5	Cognitive appraisal	Common dysfunctional thoughts contributing to perceived stress; 5 steps for cognitive reappraisal
6	Coping and resources	Typical resources and coping mechanisms for stress
7	Health	Definition of health and sense of coherence
8	Booster session	Recap of previous material

### Guidance

In order to support participants in adhering to the intervention while minimizing human capital costs, we will apply an adherence-focused guidance concept with personalized feedback on demand [for a detailed description of an application, see Refs. (38, 73, 74)]. The supportive accountability model suggests that the adherence and effectiveness of Internet-based interventions are enhanced by providing a minimum of human support by a professional seen as benevolent and competent by participants (75, 76). The guidance concept for this study was developed in line with these core elements.

Guidance for the IG will be performed by a specially trained student in a master’s program in psychology and consists of three parts: i) monitoring adherence to the intervention, ii) sending standardized motivational messages after every module, and iii) providing feedback on demand. Adherence monitoring involves personal reminders for participants who had not completed a session in the designated timeframe (7 days). Standardized motivational messages tailored to each session will be sent when participants completed one of the main modules, summarizing the content of the previous module and motivating trainees to stay engaged. Feedback on demand will be provided through the internal messaging system of the training platform, which participants may use whenever individualized content feedback is needed. Participants will then receive feedback within 48 h. If requested, participants in the IG will be able to receive automatic messages containing short, motivational prompts *via* SMS.

Participants in the active CG will receive parts (i) and (ii), but will not receive feedback on demand.

### Primary Outcome

The primary outcome is symptoms of depression as measured by the German version of the CES-D 20-item version [ADS (51); 20 items; range 0–60] at post-treatment (T1). Participants will be asked to indicate the frequency of depression-specific symptoms for a retrospective timeframe of 2 weeks on a four-point Likert scale [0 = *rarely or none of the time present* (less than 1 day), 3 = *most of the time present* (5–14 days); e.g., “During the past two weeks I had trouble keeping my mind on what I was doing.”]. Higher scores indicate greater depression severity. The CES-D shows high correlations up to *r* = 0.90 with other measures such as the Hamilton Depression Scale or the Beck Depression Inventory, and has a high retest reliability of *r* *_tt_* = 0.81, indicating the high internal validity of this instrument (51).

### Secondary Outcomes

The number of items and range of below-mentioned instruments is reported in parentheses. Unless otherwise specified, all outcomes will be measured for a retrospective timeframe of 2 weeks. For a comprehensive overview of all variables, see **Table 3**.

**Table 3:** Overview of instruments and assessment points.

Variable	Instrument	T0	T1	T2	Putative moderator
**Screening**					
Depression	CES-D	X	–	–	–
Suicidal ideation	BDI-II (Item 9)	X	–	–	–
Demographic data	–	X	–	–	–
Self-reported history of psychosis/dissociative symptoms	–	X	–	–	–
**Primary outcome**					
Depression	CES-D	X	X	X	–
**Secondary outcomes**					
*Mental health*					
Perceived stress	PSS-10	X	X	X	–
Anxiety	STAI-6	X	X	X	–
Worrying	AWQ	X	X	X	–
Emotional exhaustion	MBI-S	X	X	X	–
Behavioral activation, rumination	BADS	X	X	X	–
*Academic outcomes*					
Work impairment, productivity	PSS	X	X	X	–
College self-efficacy	CSEI	X	X	X	X
*Risk and protective factors*					
Emotion regulation skills	SEK-27	X	X	X	X
Resilience	CD-RISC	X	X	X	–
Self-compassion	SCS	X	X	X	–
Locus of control	IE-4	X	–	–	X
Self-esteem	RSES	X	X	X	–
Beliefs about stress	BASS	X	X	X	X
*Health economic measures*					
Indirect costs	TiC-P	X	X	X	–
*Health literacy and help-seeking behavior*					
Help-seeking intentions	GHSQ	X	–	–	–
Internet therapy experience	–	X	–	–	–
E-Health literacy	eHEALS	X	–	–	–
Reasons for participation	–	X	–	–	–
**Additional measures**					
Client satisfaction	CSQ-8	–	X	–	–
Intervention credibility and expectations	CEQ	X	–	–	–
Sociodemographic characteristics	–	X	–	–	X
Personality traits	BFI-10	X	–	–	X

*Mental health*. Further outcomes on mental health include perceived stress as measured by the Perceived Stress Scale 10-item version [PSS-10 (77); 10 items, range 0–40], state anxiety [short form of the Spielberger State-Trait Anxiety Inventory, STAI-6 (78, 79); 6 items, range 6–24; *at the moment*], behavioral activation, rumination and functional impairment as measured by the Behavioral Activation for Depression Scale [BADS (80); 25 items; range 0–150], and worrying [Academic Worrying Questionnaire; AWQ (81); 10 items; range 0–40]. Emotional exhaustion will be assessed using the emotional exhaustion subscale of the Maslach Burnout Inventory student version [MBI-S (82); five items, range 5–30].

*Academic outcomes*. To evaluate presenteeism and loss of productivity, the Presenteeism Scale for Students’ [PSS (83)] subscale for work impairment (Work Impairment Scale; WIS; 10 items; range 10–50) will be administered. The WIS is based on the Stanford Presenteeism Scale [SPS-6 (84, 85)] and allows to assess work-related outcomes in non-occupational samples (86, 87). Productivity losses will be assessed by an adaption of the PSS’ work output scale (WOS) investigating the percentage to which participants are able to reach their usual academic productivity. Productivity will be rated on a visual analog scale ranging from 0% = *completely unproductive* to 100% = *full productivity*. College self-efficacy will be assessed by the College Self-Efficacy Inventory [CSEI (88); 13 items; range 13–65].

*Modifiable risk and protective factors*. Risk and protective factors for mental illness include resilience as measured by the Connor-Davidson Resilience Scale short form [CD-RISC (89); 2 items; range 0–8], emotion regulation competencies [German version of the Assessment of Emotion Regulation Skills; SEK-27; State Version (90); 27 items; range 27–108], self-compassion [Self-Compassion Scale; SCS-D (91); 12 items; range 12–60], self-esteem as measured by the Rosenberg Self-Esteem Scale [RSES (92); 10 items; range 10–40], and locus of control as measured by the Short Scale for the Assessment of Locus of Control [IE-4 (93)]. The IE-4 consists of two subscales for internal and external locus of control, each consisting of two items (range 2–10). Personal beliefs about the controllability and the harmful and positive nature of stress will be assessed using the Beliefs about Stress Scales’ [BASS (94)] subscales for positive (four items; range 4–16), negative (eight items; range 8–32), and controllability beliefs (three items; range 3–12).

*Health economic measures*. Indirect costs due to presenteeism and absenteeism will be assessed using the productivity loss subscale of the Trimbos/iMTA questionnaire for costs associated with psychiatric illness [TiC-P (95)], which was adapted to the context of distant-learning students in tertiary education. With this questionnaire, participants register approximations of their working hours at university, household work, and monthly salaries when holding a job besides their studies. Indirect costs, such as the number of “work loss” days (absenteeism from work) or the number of “work cut-back” days (reduced productivity at work), can be assessed.

*Health literacy and help-seeking intentions*. To assess the help-seeking preferences of participants, the General Help-Seeking Questionnaire for personal–emotional problems [GHSQ-Per-Emot (96); seven items; range 7–77] will be administered at baseline. E-Health literacy will be assessed at baseline using the German E-Health Literacy Scale [eHEALS (97); eight items; range 8–40]. Online counseling experiences and awareness will be assessed by two items extracted from the SOEP-IS module “Internet-based psychotherapy” (98) (“have you ever utilized psychosocial or psychotherapeutic online counselling before?”; “have you ever heard of or read about Internet(-based) psychotherapies?”; yes/no). A self-constructed questionnaire will be used to assess the reasons for participating in the intervention, following the help-seeking behavior model by Rickwood, Thomas & Bradford (99).

*Additional measures*. Additional questionnaires will assess demographic variables and client satisfaction with the intervention [German version of the Client Satisfaction Questionnaire, adapted to the online context; CSQ-8; (100, 101); eight items]. Personality traits will be assessed at baseline using the 10-item Big Five Inventory [BFI-10 (102); 10 items; range 2–10 per dimension]. Treatment credibility and expectancies will be measured at baseline by administering the Credibility and Expectancy Questionnaire [CEQ (103); six items]; results for each subscale incorporated in this questionnaire will be transformed into a 100-point overall score for each participant. Participants in the IG will have the opportunity to suggest intervention improvements after every session and give feedback on each modules’ usefulness (0 = *not useful at all*, 4 = *very useful*), complexity (0 = *very complex*, 4 = *very easy*), and duration until termination (0 = *less than ½ hour*, 4 = *more than 1½ hours*) on a five-point Likert scale.

*Moderator variables*. Following variables mentioned above will be analyzed in explorative analysis of putative moderators: i) emotion regulation skills as measured by the SEK-27, ii) personality traits as assessed by the BFI-10, iii) locus of control as measured by the IE-4, iv) academic self-efficacy (CSEI), v) beliefs about stress (BASS), and vi) demographic variables. These variables were selected based on a hypothesis-generating approach, using a broad variety of possible moderators at baseline in order to detect putative moderators for sufficiently powered future studies (104).

### Statistical Analyses

*Clinical evaluation*. Analyses based on the ITT principle will be conducted, with missing data imputed using a Markov chain Monte Carlo multivariate imputation algorithm with 100 estimations per missing.

The hypothesized superiority of the Internet-based stress intervention will be tested with regard to i) change in participants’ scores of depression and secondary outcomes from baseline (T0) to post-intervention (T1) and 3-month follow-up (T2), ii) the amount of participants with treatment response, iii) the amount of participants who experienced symptom deterioration throughout the trial, and iv) the number of individuals who achieved clinically relevant amelioration of symptoms in the IG compared to the CG.

Differences in the change of symptoms between study arms will be assessed using univariate analysis of covariance with scores at baseline used as covariate. Changes in within-group scores between baseline, T2, and T3 will be explored. Effect sizes (Cohen’s *d*) will be calculated based on the imputed data set for between- and within-group changes with 95% CIs (105). To mitigate the risk of type I error inflation due to multiple testing, *p*-values will be adjusted using Holm’s method (106).

To ascertain the number of participants attaining a reliable improvement in depressive symptomatology, participants will be coded as responders or non-responders according to the widely used *Reliable Change Index* [RCI (107, 108)]. To assess the clinically relevant impact on depression for the IG compared to control, we will calculate and compare the number of participants who achieved a reduction of >50% in depressive symptoms compared to baseline at both T1 and T2.

Negative effects of the intervention will be evaluated by the number of participants with reliable deterioration concerning depression scores through the RCI.

*Moderators of treatment effects*. To assess the effect of putative moderators on changes in outcomes (i.e., T0 – T1), an ordinary least squares (OLS) regression-based framework for estimating interactions in moderation models will be used, along with simple slopes for probing interactions. In order to test the robustness of our findings and to examine differences between imputed data and complete cases, separate sensitivity analyses (109, 110) will be conducted. Moreover, to assess relevant subgroup effects, follow-up simple slope analyses for possible significant interaction effects will be conducted. The slope and significance of the intervention main effect will be evaluated for conditional values (one standard deviation below and one standard deviation above the mean) of the moderator (111). For more precise information on conditional effects of the moderators, the Johnson–Neyman technique will be applied (112).

*Cost–benefit evaluation*. According with common practice, a probabilistic approach will be used to make health-economic inferences (113). The total benefit of the intervention will be derived by subtracting productivity loss costs assessed by the TiC-P of the CG from the ones of the IG. The cost–benefit relationship will be reported by calculating the three most frequently used metrics: i) the net benefits (NB), the net monetary gain of the intervention; ii) the benefit–cost ratio (BCR), the money saved for one monetary unit invested; and iii) the return-on-investment (ROI), indicating the percentage of profit with every monetary unit invested. Bootstrapped 0.95% CIs with 2,500 replications will be estimated around these metrics.

Sensitivity analyses will be conducted to assess the robustness of the findings and to account for uncertainty regarding the actual prize of implementing the intervention into health care. Further health economic analyses will be conducted if feasible.

*Help-seeking behavior analysis*. To make assertions on the help-seeking intentions and behaviors of participants, baseline data of all participants from the GHSQ-Per-Emot (96), eHEALS (97), “awareness of online counseling,” and “reasons for participation” questionnaires will be analyzed using descriptive statistics.

*Process evaluation*. Descriptive statistics will be used for process evaluation. To assess overall user satisfaction across various domains, CSQ-8 item data will be examined. Acceptance of intervention modules will be analyzed using the module feedback of the IG. Adherence will be assessed by analyzing intervention completion rates tracked within the intervention platform.

## Discussion

This protocol describes the rationale and design of a two-armed RCT evaluating the effectiveness, moderators, and health economic outcomes of an Internet- and App-based stress intervention for distance-learning students. We hypothesize the intervention to be more effective in reducing symptoms of depression and improving other mental health-related outcomes when compared to a CG receiving Internet-based psychoeducation.

The relevance of early intervention in mental health promotion and prevention has become increasingly evident in recent years (42, 114). Easily accessible and acceptable instruments are urgently needed when facing discouragingly low utilization rates found for conventional health promotion approaches directed at the general public (115). This trial will contribute to the ongoing investigation into the potential of Internet-based interventions to reach out to individuals who would not otherwise seek treatment due to psychological barriers. Furthermore, this study will explore moderators of intervention effects and assess the intervention’s health economic potential when implemented into routine care. Help-seeking behavior will be assessed to attain more knowledge on the causes and motivation of distance-learning students when seeking help *via* the Internet.

This trial will employ a cutoff for elevated symptoms of depression, although not restricting eligibility to individuals showing symptoms of full-blown depression only. Although in its content, the intervention at hand focuses on academic stress, we did not decide for a cutoff for perceived stress, as this trial is aimed at evaluating the intervention’s effectiveness as an intervention instrument in individuals with an indicated risk for depression and in individuals potentially suffering from major depression. Perceived stress has been associated with the development (33) and worse trajectories (34, 35) of clinical depression. The potential of Internet-based stress interventions as an early intervention and treatment instrument in depressive target groups, however, has not yet been evaluated. Mental health stigma, a known barrier to treatment utilization, has been shown to be greatest among highly distressed individuals with low depression literacy (116). If the intervention evaluated in this trial is found to be effective, Internet-based stress interventions could be investigated as a potential mean to address depression in individuals for whom a conventional mental health disorder treatment would not be an option.

We will compare the effects of the intervention to an Internet-based psychoeducation program of similar length. Meta-analytic evidence suggests that Internet-based interventions for college students show moderate to large effect sizes when compared to a waiting CG. This superiority, however, could not be ascertained when compared to active CGs (117). Treatment expectancies have been discussed as an artifact in clinical evaluation studies utilizing waitlist CGs. Participants waiting for treatment may be less motivated to initiate health-related behavior changes (118–120), which could over-accentuate effects attributed to an intervention (120, 121). Findings in this trial will provide the opportunity to investigate the effects of an Internet-based intervention employing evidence-based cognitive–behavioral techniques compared to a CG in which participants are also actively engaged.

Findings of this trial will have some limitations. First, an open recruitment strategy is employed, in which potential participants are actively recruited from the student population. Therefore, we cannot rule out selection bias, with more motivated individuals attaining access to the study. Second, due to feasibility reasons, this trial is somewhat underpowered to reliably detect significant treatment moderators. This might impede finding statistically significant factors contributing to this interventions’ effectiveness that would have been detected in a larger study sample (122, 123). Same applies to health-economic inferences, for which a probabilistic approach will be applied in this study. Third, due to logistic reasons, this trial will only use self-administered questionnaires and will not assess diagnostic status or physiological measures.

There is a growing number of studies investigating the efficacy of Internet-based interventions in reducing various symptoms of mental illness (124, 125). Much less is known, however, on the potential of such interventions in distance-learning student samples, and their impact on academic functioning, for which this study will provide tangible empirical results. Results will also provide the opportunity to investigate the practicability of Internet-based stress interventions as an approach to address mental health issues in individuals with subthreshold or full-blown symptoms of depression and increase their use of health services.

### Trial Status

Recruitment started in April 2017. Follow-up assessments for the last participant are expected to be completed by March 2019.

## Ethics Statement

All procedures involved in the study are consistent with the generally accepted standards of ethical practice. All potential participants have to provide written informed consent to be eligible for study inclusion. The study was approved by the University of Erlangen-Nuremberg ethics committee (Erlangen, Germany; 33_17 Bc).

## Author Contributions

DE and JA-H initiated the study. MH, LF, MD, LK, KB, CS, A-CZ, HB, and DL made relevant contributions to the study design and procedure. MH wrote the initial draft of the manuscript. All authors read and approved the final manuscript.

## Funding

The study was funded through internal research funds of the *Fern Universität in Hagen*.

## Conflict of Interest Statement

DE is a stakeholder of the “Institute for Online Health Trainings”, which aims to transfer scientific knowledge related to the present research into routine health care. The remaining authors declare that the research was conducted in the absence of any commercial or financial relationships that could be construed as a potential conflict of interest.

## Abbreviations

CG, control group; CONSORT, Consolidated Standards of Reporting Trials; IG, intervention group; ITT, intention to treat; RCI, Reliable Change Index; RCT, randomized controlled trial; SPIRIT, Standard Protocol Items: Recommendations for Intervention Trials; T0, baseline; T1, post-assessment (7 weeks after randomization); T2, 3-month follow-up (3 months after randomization).

## Supplementary Material

Table S1 SPIRIT Checklist.

Figure S1 SPIRIT Figure.

## References

[1] Auerbach RP Alonso J Axinn WG Cuijpers P Ebert DD Green JG Mental disorders among college students in the World Health Organization World Mental Health Surveys. Psychol Med (2016) 46(14):1–16. 10.1017/S0033291716001665 27484622 PMC5129654

[2] Blanco C Okuda M Wright C Hasin DS Grant BF Liu S-M Mental health of college students and their non–college-attending peers. Arch Gen Psychiatry (2008) 65(12):1429. 10.1001/archpsyc.65.12.1429 19047530 PMC2734947

[3] Bruffaerts R Mortier P Kiekens G Auerbach RP Cuijpersd P Demyttenaere K Mental health problems in college freshmen: prevalence and academic functioning. J Affect Disord (2017). 225:97–103. 10.1016/j.jad.2017.07.044 28802728 PMC5846318

[4] Auerbach RP Mortier P Bruffaerts R Alonso J Benjet C Cuijpers P WHO World Mental Health Surveys International College Student Project: prevalence and distribution of mental disorders. J Abnorm Psychol (2018). 127(7):623–38. 10.1037/abn0000362 PMC6193834 30211576

[5] Ishii T Tachikawa H Shiratori Y Hori T Aiba M Kuga K What kinds of factors affect the academic outcomes of university students with mental disorders? A retrospective study based on medical records. Asian J Psychiatry (2018) 32:67–72. 10.1016/j.ajp.2017.11.017 29216609

[6] Eisenberg D Golberstein E Hunt JB Mental health and academic success in college. BE J Econ Anal Policy (2009) 9(1). 10.2202/1935-1682.2191

[7] Hysenbegasi A Hass SL Rowland CR The impact of depression on the academic productivity of university students. J Ment Health Policy Econ (2005) 8(3):145. 16278502

[8] Kessler RC Foster CL Saunders WB Stang PE Social consequences of psychiatric disorders, I: Educational attainment. Am J Psychiatry (1995) 152(7):1026. 10.1176/ajp.152.7.1026 7793438

[9] Breslau J Lane M Sampson N Kessler RC Mental disorders and subsequent educational attainment in a US national sample. J Psychiatr Res (2008) 42(9):708–16. 10.1016/j.jpsychires.2008.01.016 PMC2748981 18331741

[10] Scott KM Lim C Al-Hamzawi A Alonso J Bruffaerts R Caldas-de-Almeida JM Association of mental disorders with subsequent chronic physical conditions: world mental health surveys from 17 countries. JAMA Psychiatry (2016) 73(2):150–8. 10.1001/jamapsychiatry.2015.2688 PMC5333921 26719969

[11] Niederkrotenthaler T Tinghög P Alexanderson K Dahlin M Wang M Beckman K Future risk of labour market marginalization in young suicide attempters—a population-based prospective cohort study. Int J Epidemiol (2014) 43(5):1520–30. 10.1093/ije/dyu155 25102855

[12] Goldman-Mellor SJ Caspi A Harrington H Hogan S Nada-Raja S Poulton R Suicide attempt in young people: a signal for long-term health care and social needs. JAMA Psychiatry (2014) 71(2):119–27. 10.1001/jamapsychiatry.2013.2803 PMC3946312 24306041

[13] Bostwick JM Pankratz VS Affective disorders and suicide risk: a reexamination. Am J Psychiatry (2000) 157(12):1925–32. 10.1176/appi.ajp.157.12.1925 11097952

[14] Mortier P Demyttenaere K Auerbach RP Cuijpers P Green JG Kiekens G First onset of suicidal thoughts and behaviours in college. J Affect Disord (2017) 207(9):291–9. 10.1016/j.jad.2016.09.033 PMC5460371 27741465

[15] Mortier P Auerbach RP Alonso J Bantjes J Benjet C Cuijpers P Suicidal thoughts and behaviors among first-year college students: results from the WMH-ICS Project. J Am Acad Child Adolesc Psychiatry (2018) 57(4):263–73. 10.1016/j.jaac.2018.01.018 PMC6444360 29588052

[16] Mortier P Auerbach RP Alonso J Axinn WG Cuijpers P Ebert DD Suicidal thoughts and behaviors among college students and same-aged peers: results from the World Health Organization World Mental Health Surveys. Soc Psychiatry Psychiatr Epidemiol (2018) 53(3):1–10. 10.1007/s00127-018-1481-6 29340781 PMC5896296

[17] Turner JC Leno EV Keller A Causes of mortality among American college students: a pilot study. J Coll Stud Psychother (2013) 27(1):31–42. 10.1080/87568225.2013.739022 PMC4535338 26322333

[18] Simpson O Supporting students for success in online and distance education. New York: Routledge (2012). ISBN:9780415509107 10.4324/9780203095737

[19] Stoessel K Ihme TA Barbarino M-L Fisseler B Stürmer S Sociodemographic diversity and distance education: who drops out from academic programs and why? Res High Educ (2015) 56(3):228–46. 10.1007/s11162-014-9343-x

[20] Apolinário-Hagen J Groenewold SD Fritsche L Kemper J Krings L Salewski C Die Gesundheit Fernstudierender stärken. Prävent Gesundheitsförderung (2017) 13(2):1–8. 10.1007/s11553-017-0620-3

[21] Richardson JTE Academic Attainment in Students with Mental Health Difficulties in Distance Education. Int J Ment Health (2015) 44(3):231–40. 10.1080/00207411.2015.1035084

[22] Eisenberg D Downs MF Golberstein E Zivin K Stigma and help seeking for mental health among college students. Med Care Res Rev (2009) 66(5):522–41. 10.1177/1077558709335173 19454625

[23] Andrade LH Alonso J Mneimneh Z Wells JE Al-Hamzawi A Borges G Barriers to mental health treatment: results from the WHO World Mental Health surveys. Psychol Med (2014) 44(6):1303–17. 10.1017/S0033291713001943 PMC4100460 23931656

[24] Ebert DD Van Daele T Nordgreen T Karekla M Compare AT Zarbo C Internet- and mobile-based psychological interventions: applications, efficacy, and potential for improving mental health. Eur Psychol (2018) 23:167–87. 10.1027/1016-9040/a000318

[25] Ebert DD Cuijpers P Muñoz RF Baumeister H Prevention of mental health disorders using internet-and mobile-based interventions: a narrative review and recommendations for future research. Front Psychiatry (2017) 8:116. 10.3389/fpsyt.2017.00116 28848454 PMC5554359

[26] Davies EB Morriss R Glazebrook C Computer-delivered and web-based interventions to improve depression, anxiety, and psychological well-being of university students: a systematic review and meta-analysis. J Med Internet Res (2014) 16(5):1–22. 10.2196/jmir.3142 PMC4051748 24836465

[27] Heber E Ebert DD Lehr D Cuijpers P Berking M Nobis S The benefit of web- and computer-based interventions for stress: a systematic review and meta-analysis. J Med Internet Res (2017) 19(2):e32. 10.2196/jmir.5774 28213341 PMC5336602

[28] Ebert DD Gollwitzer M Riper H Cuijpers P Baumeister H Berking M For whom does it work? moderators of outcome on the effect of a transdiagnostic internet-based maintenance treatment after inpatient psychotherapy: randomized controlled trial. J Med Internet Res (2013) 15(10):e191. 10.2196/jmir.2511 24113764 PMC3849694

[29] Lindefors N Andersson G Guided Internet-based treatments in psychiatry. Cham, Switzerland: Springer International Publishing (2016). ISBN:9783319060828 10.1007/978-3-319-06083-5

[30] Ryan ML Shochet IM Stallman HM Universal online interventions might engage psychologically distressed university students who are unlikely to seek formal help. Adv Ment Health (2010) 9(1):73–83. 10.5172/jamh.9.1.73

[31] Apolinário-Hagen J Vehreschild V Alkoudmani RM Current views and perspectives on e-mental health: an exploratory survey study for understanding public attitudes toward internet-based psychotherapy in Germany. JMIR Ment Health (2017) 4(1):e8. 10.2196/mental.6375 28232298 PMC5378055

[32] Harrer M Adam SH Fleischmann RJ Baumeister H Auerbach RP Bruffaerts R Effectiveness of an Internet- and App-based intervention for college students with elevated stress: results of a randomized controlled trial (in press). J Med Internet Res (2018) 20(4):e136. 10.2196/jmir.9293 29685870 PMC5938594

[33] Cohen S Janicki-Deverts D Miller GE Psychological stress and disease. JAMA (2007) 298(14):1685–7. 10.1001/jama.298.14.1685 17925521

[34] Mazure CM Life stressors as risk factors in depression. Clin Psychol Sci Pract (1998) 5(3):291–313. 10.1111/j.1468-2850.1998.tb00151.x

[35] Hammen C Stress and depression. Annu Rev Clin Psychol (2005) 1:293–319. 10.1146/annurev.clinpsy.1.102803.143938 17716090

[36] Ebert DD Heber E Berking M Riper H Cuijpers P Funk B Self-guided internet-based and mobile-based stress management for employees: results of a randomised controlled trial. Occup Environ Med (2016) 73(5):315–23. 10.1136/oemed-2015-103269 26884049

[37] Heber E Lehr D Ebert DD Berking M Riper H Web-based and mobile stress management intervention for employees: a randomized controlled trial. J Med Internet Res (2016) 18(1):e21. 10.2196/jmir.5112 26818683 PMC4749847

[38] Ebert DD Lehr D Heber E Riper H Cuijpers P Berking M Internet- and mobile-based stress management for employees with adherence-focused guidance: efficacy and mechanism of change. Scand J Work Environ Health (2016) 42(5):382–94. 10.5271/sjweh.3573 27249161

[39] Weisel KK Lehr D Heber E Zarski A-C Berking M Riper H Severely burdened individuals do not need to be excluded from internet-based and mobile-based stress management: effect modifiers of treatment outcomes from three randomized controlled trials. J Med Internet Res (2018) 20(6):e211. 10.2196/jmir.9387 29921562 PMC6030574

[40] Barth J Munder T Gerger H Nüesch E Trelle S Znoj H Comparative efficacy of seven psychotherapeutic interventions for patients with depression: a network meta-analysis. Focus (2016) 14(2):229–43. 10.1176/appi.focus.140201 PMC6519647 31997951

[41] Buntrock C Ebert DD Lehr D Smit F Riper H Berking M Effect of a web-based guided self-help intervention for prevention of major depression in adults with subthreshold depression. JAMA (2016) 315(17):1854. 10.1001/jama.2016.4326 27139058

[42] Ebert DD Cuijpers P Muñoz RF Baumeister H Prevention of Mental Health Disorders using Internet and mobile-based Interventions: a narrative review and recommendations for future research. Front Psychiatry Frontiers (2017) 8:116. 10.3389/fpsyt.2017.00116 PMC5554359 28848454

[43] Königbauer J Letsch J Doebler P Ebert D Baumeister H Internet- and mobile-based depression interventions for people with diagnosed depression: a systematic review and meta-analysis. J Affect Disord (2017) 223:28–40. 10.1016/j.jad.2017.07.021 28715726

[44] Ebert DD Cuijpers P It is time to invest in the prevention of depression. JAMA Netw Open (2018) 1(2):e180335–e180335. 10.1001/jamanetworkopen.2018.0335 30646081

[45] Kessler RC The potential of predictive analytics to provide clinical decision support in depression treatment planning. Curr Opin Psychiatry (2018) 31(1):32–9. 10.1097/YCO.0000000000000377 29076894

[46] Ebert DD Buntrock C Reins JA Zimmermann J Cuijpers P Efficacy and moderators of psychological interventions in treating subclinical symptoms of depression and preventing major depressive disorder onsets: protocol for an individual patient data meta-analysis of randomised controlled trials. BMJ Open (2018) 8(3):e018582 10.1136/bmjopen-2017-018582 PMC5857689 29549201

[47] Kessler RC Van Loo HM Wardenaar KJ Bossarte RM Brenner LA Ebert DD Using patient self-reports to study heterogeneity of treatment effects in major depressive disorder. Epidemiol Psychiatr Sci (2017) 26(1):22–36. 10.1017/S2045796016000020 26810628 PMC5125904

[48] Altman DG Schulz KF Moher D Egger M Davidoff F Elbourne D The revised CONSORT statement for reporting randomized trials: explanation and elaboration. Ann Intern Med (2011) 134(8):663–94. 10.7326/0003-4819-134-8-200104170-00012 11304107

[49] Proudfoot J Klein B Barak A Carlbring P Cuijpers P Lange A Establishing guidelines for executing and reporting internet intervention research. Cogn Behav Ther (2011) 40(2):82–97. 10.1080/16506073.2011.573807 25155812

[50] Chan A-W Tetzlaff JM Altman DG Laupacis A Gøtzsche PC Krleža-Jerić K SPIRIT 2013 statement: defining standard protocol items for clinical trials. Ann Intern Med (2013) 158(3):200–7. 10.7326/0003-4819-158-3-201302050-00583 PMC5114123 23295957

[51] Hautzinger M Bailer M Hofmeister D Keller F Center for Epidemiological Studies Depression Scale (CES-D; Radloff, L. Psychiatr Prax (2012) 39(6):302–4. 10.1055/s-0032-1326702

[52] Hautzinger M Keller F Kühner C Beck Depressions-Inventar (BDI-II). In: Harcourt Test Services Frankfurt. Frankfurt/Main: Harcourt Test Services (2006)

[53] DGPPN , BÄK , KBV , AWMF eds. Leitliniengruppe Unipolare Depression. In: S3-Guideline/Nationale VersorgungsLeitlinie Unipolar Depression. 10.6101/AZQ/000364

[54] Faul F Erdfelder E Lang A-G Buchner A G * Power 3: a flexible statistical power analysis program for the social, behavioral, and biomedical sciences. Behav Res Methods (2007) 39(2):175–91. 10.3758/BF03193146 17695343

[55] Hintz S Frazier PA Meredith L Evaluating an online stress management intervention for college students. J Couns Psychol (2015) 62(2):137–47. 10.1037/cou0000014 24635586

[56] Chiauzzi E Brevard J Thum C Decembrele S Lord S MyStudentBody-Stress: an online stress management intervention for college students. J Health Commun (2008) Aug 2813(6):555–72. 10.1080/10810730802281668 18726812

[57] Cavanagh K Strauss C Cicconi F Griffiths N Wyper A Jones F A randomised controlled trial of a brief online mindfulness-based intervention. Behav Res Ther (2013) 51(9):573–8. 10.1016/j.brat.2013.06.003 23872699

[58] Frazier P Meredith L Greer C Paulsen JA Howard K Dietz LR Randomized controlled trial evaluating the effectiveness of a web-based stress management program among community college students. Anxiety Stress Coping (2015) Sep 328(5):576–86. 10.1080/10615806.2014.987666 25420030

[59] Day V McGrath P Wojtowicz M Internet-based guided self-help for university students with anxiety, depression and stress: a randomized controlled clinical trial. Behav Res Ther (2013) 51(7):344–51. 10.1016/j.brat.2013.03.003 23639300

[60] Fleischmann RJ Harrer M Zarski A-C Baumeister H Lehr D Ebert DD Patients’ experiences in a guided Internet- and App-based stress intervention for college students: a qualitative study. Internet Interv (2018) 12:130–40. 10.1016/j.invent.2017.12.001 PMC6096317 30135777

[61] Lazarus RS Folkman S Stress, appraisal, and coping. New York: Springer (1984), ISBN: 0-8261-4191-9

[62] Weiten W Dunn DS Hammer EY Psychology applied to modern life: adjustment in the 21st century. 11th ed Stamford: Cengage (2016).

[63] Berking M Wupperman P Emotion regulation and mental health: recent findings, current challenges, and future directions. Curr Opin Psychiatry (2012) 25(2):128–34. 10.1097/YCO.0b013e3283503669 22262030

[64] Malouff JM Thorsteinsson EB Schutte NS The efficacy of problem solving therapy in reducing mental and physical health problems: a meta-analysis. Clin Psychol Rev (2007) 27(1):46–57. 10.1016/j.cpr.2005.12.005 16480801

[65] Bell AC D’Zurilla TJ Problem-solving therapy for depression: a meta-analysis. Clin Psychol Rev (2009) 29(4):348–53. 10.1016/j.cpr.2009.02.003 19299058

[66] Wirtz CM Radkovsky A Ebert DD Berking M Successful application of adaptive emotion regulation skills predicts the subsequent reduction of depressive symptom severity but neither the reduction of anxiety nor the reduction of general distress during the treatment of Major depressive disorder. PLoS ONE (2014) 9(10):e108288. 10.1371/journal.pone.0108288 25330159 PMC4203678

[67] Ehring T Tuschen-Caffier B Schnülle J Fischer S Gross JJ Emotion regulation and vulnerability to depression: spontaneous versus instructed use of emotion suppression and reappraisal. Emotion (2010) 10(4):563–72. 10.1037/a0019010 20677873

[68] Henry JD Castellini J Moses E Scott JG Emotion regulation in adolescents with mental health problems. J Clin Exp Neuropsychol (2015) 38(2):197–207. 10.1080/13803395.2015.1100276 26594853

[69] Hopfinger L Berking M Bockting CLH Ebert DD Emotion regulation protects against recurrence of depressive symptoms following inpatient care for major depressive disorder. Behav Ther (2017) 48(6):739–49. 10.1016/j.beth.2017.03.003 29029672

[70] Berking M Ebert D Cuijpers P Hofmann SG Emotion regulation skills training enhances the efficacy of inpatient cognitive behavioral therapy for major depressive disorder: a randomized controlled trial. Psychother Psychosom (2013) 82(4):234–45. 10.1159/000348448 23712210

[71] Berking M Meier C Wupperman P Enhancing emotion-regulation skills in police officers: results of a pilot controlled study. Behav Ther (2010) 41(3):329–39. 10.1016/j.beth.2009.08.001 20569782

[72] Gross JJ Emotion regulation: current status and future prospects. Psychol Inq (2015) 26(1):1–26. 10.1080/1047840X.2014.940781

[73] Ebert DD Lehr D Smit F Zarski AC Riper H Heber E Efficacy and cost-effectiveness of minimal guided and unguided internet-based mobile supported stress-management in employees with occupational stress: a three-armed randomised controlled trial. BMC Public Health (2014) 14(807):1471–2458. 10.1186/1471-2458-14-807 PMC4153891 25099533

[74] Zarski A-C Lehr D Berking M Riper H Cuijpers P Ebert DD Adherence to internet-based mobile-supported stress management: a pooled analysis of individual participant data from three randomized controlled trials. J Med Internet Res (2016) 18(6):e146. 10.2196/jmir.4493 27357528 PMC4945816

[75] Mohr DC Cuijpers P Lehman K Supportive accountability: a model for providing human support to enhance adherence to eHealth interventions. J Med Internet Res (2011) 13(1):e30. 10.2196/jmir.1602 21393123 PMC3221353

[76] Schueller SM Tomasino KN Mohr DC Integrating human support into behavioral intervention technologies: the efficiency model of support. Clin Psychol Sci Pract (2016) 24(1):27–45. 10.1111/cpsp.12173

[77] Cohen S Kamarck T Mermelstein R A global measure of perceived stress. J Health Soc Behav (1983) 24(4):385–96. 10.2307/2136404 6668417

[78] Marteau TM Bekker H The development of a six-item short-form of the state scale of the Spielberger State-Trait Anxiety Inventory (STAI). Br J Clin Psychol (1992) 31(3):301–6. 10.1111/j.2044-8260.1992.tb00997.x 1393159

[79] Laux L Glanzmann P Schaffner P Spielberger C Das State-Trait-Anxiety-Inventory (STAI): Theoretische Grundlagen und Handanweisung. Weinheim: Beltz (1981).

[80] Fuhr K Hautzinger M Krisch K Berking M Ebert DD Validation of the Behavioral Activation for Depression Scale (BADS)—Psychometric properties of the long and short form. Compr Psychiatry (2016) 66:209–18. 10.1016/j.comppsych.2016.02.004 26995255

[81] Wolitzky-Taylor K Telch M Efficacy of self-administered treatments for pathological academic worry: a randomized controlled trial. Behav Res Ther (2010) 48(9):840–50. 10.1016/j.brat.2010.03.019 20663491

[82] Gumz A Erices R Brähler E Zenger M Factorial structure and psychometric criteria of the German translation of the Maslach Burnout Inventory—Student Version by Schaufeli et al. (MBI-SS). Psychother Psychosom Med Psychol (2013) 63(2):77–84. 10.1055/s-0032-1323695 23408302

[83] Matsushita M Adachi H Arakida M Namura I Takahashi Y Miyata M Presenteeism in college students: reliability and validity of the presenteeism scale for students. Qual Life Res (2011) 20(3):439–46. 10.1007/s11136-010-9763-9 20945160

[84] Turpin RS Ozminkowski RJ Sharda CE Collins JJ Berger ML Billotti GM Reliability and validity of the Stanford presenteeism scale. J Occup Environ Med (2004) 46(11):1123–33. 10.1097/01.jom.0000144999.35675.a0 15534499

[85] Yamashita M Arakida M Reliability and validity of the Japanese version of the Stanford Presenteeism Scale in female employees at 2 Japanese enterprises. J Occup Health (2008) 50(1):66–69. 10.1539/joh.50.66 18285647

[86] Mikami A Matsushita M Adachi H Suganuma N Koyama A Ichimi N Sense of coherence, health problems, and presenteeism in Japanese university students. Asian J Psychiatry (2013) 6(5):369–72. 10.1016/j.ajp.2013.03.008 24011682

[87] Ferritto VR Maritime education factors and presenteeism: a comparative quantitative study. WMU J Marit Aff (2016) 15(2):353–80. 10.1007/s13437-015-0098-9

[88] Solberg VS O’Brien K Villareal P Kennel R Davis B Self-efficacy and Hispanic college students: validation of the college self-efficacy instrument. Hisp J Behav Sci (1993) 15:80–95. 10.1177/07399863930151004

[89] Connor KM Davidson JRT Development of a new Resilience scale: the Connor-Davidson Resilience scale (CD-RISC). Depress Anxiety (2003) 18(2):76–82. 10.1002/da.10113 12964174

[90] Berking M Znoj H Entwicklung und validierung eines fragebogens zur standardisierten selbsteinschätzung emotionaler kompetenzen (SEK-27). Z Psychiatr Psychol Psychother (2008) 56(2):141–53. 10.1024/1661-4747.56.2.141

[91] Hupfeld J Ruffieux N Validation of a German version of the Self-Compassion Scale (SCS-D) Abstract. Z Klin Psychol Psychother (2011) 40(2):115–23. 10.1026/1616-3443/a000088

[92] Ferring D Filipp SH Measurement of self-esteem: findings on reliability, validity, and stability of the Rosenberg Scale. Diagnostica (1996) 42:292.

[93] Kovaleva A Beierlein C Kemper CJ Rammstedt B Eine Kurzskala zur Messung von Kontrollüberzeugung: die Skala Internale- Externale-Kontrollüberzeugung-4 (IE-4)s. Mannheim : GESIS – Leibniz-Institut für Sozialwissenschaften (2012). Report No.: 2012/19

[94] Laferton JAC Stenzel NM Fischer S The Beliefs About Stress Scale (BASS): development, reliability, and validity. Int J Stress Manag (2016) 25(1):72–83. 10.1037/str0000047

[95] Bouwmans C De Jong K Timman R Zijlstra-Vlasveld M Van der Feltz-Cornelis C Tan SS Feasibility, reliability and validity of a questionnaire on healthcare consumption and productivity loss in patients with a psychiatric disorder (TiC-P). BMC Health Serv Res (2013) 13(1):217. 10.1186/1472-6963-13-217 23768141 PMC3694473

[96] Wilson CJ Deane FP Ciarrochi J Rickwood D Measuring help-seeking intentions: properties of the general help-seeking questionnaire. Can J Couns (2005) 39(1):15–28. 10.1037/t42876-000

[97] Soellner R Huber S Reder M The concept of eHealth literacy and its measurement. J Media Psychol (2014) 26:29–38. 10.1027/1864-1105/a000104

[98] DIW (2016). SOEP Wave Report 2016.

[99] Rickwood D Thomas K Bradford S Help-seeking measures in mental health: a rapid review. Sax Inst (2012): 1–35.

[100] Nguyen TD Attkisson CC Stegner BL Assessment of patient satisfaction: development and refinement of a service evaluation questionnaire. Eval Program Plann (1983) 6(3–4):299–313. 10.1016/0149-7189(83)90010-1 10267258

[101] Boß L Lehr D Reis D Vis C Riper H Berking M Reliability and validity of assessing user satisfaction with web-based health interventions. J Med Internet Res (2016) 18(8):e234. 10.2196/jmir.5952 27582341 PMC5023944

[102] Rammstedt B Kemper C Klein MC Beierlein C Kovaleva A Eine kurze Skala zur Messung der fünf Dimensionen der Persönlichkeit: Big-Five-Inventory-10 (BFI-10). Methoden Daten Anal Mda (2013) 7(2):233–49. 10.12758/mda.2013.013

[103] Devilly GJ Borkovec TD Psychometric properties of the credibility/expectancy questionnaire. J Behav Ther Exp Psychiatry (2000) 31(2):73–86. 10.1016/S0005-7916(00)00012-4 11132119

[104] Kraemer HC Wilson GT Fairburn CG Agras WS Mediators and moderators of treatment effects in randomized clinical trials. Arch Gen Psychiatry (2002) 59(10):877–83. 10.1001/archpsyc.59.10.877 12365874

[105] Hedges LV Olkin I Statistical methods for meta-analysis. Orlando: Academic Press (2014), ISBN: 9780123363800 [ https://www.elsevier.com/books/statistical-methods-for-meta-analysis/hedges/978-0-08-057065-5]

[106] Holm S A simple sequentially rejective multiple test procedure. Scand J Stat (1979) 6(2):65–70. 10.2307/4615733

[107] Jacobson NS Truax P Clinical significance: a statistical approach to defining meaningful change in psychotherapy research. J Consult Clin Psychol (1991) 59(1):12–9. 10.1037//0022-006X.59.1.12 2002127

[108] Zahra D Hedge C The reliable change index: why isn’t it more popular in academic psychology? Psychol Postgrad Aff Group Q (2010) 76:14–9.

[109] Thabane L Mbuagbaw L Zhang S Samaan Z Marcucci M Ye C A tutorial on sensitivity analyses in clinical trials: the what, why, when and how. BMC Med Res Methodol (2013) 13(1):92. 10.1186/1471-2288-13-92 23855337 PMC3720188

[110] Wright CC Sim J Intention-to-treat approach to data from randomized controlled trials: a sensitivity analysis. J Clin Epidemiol (2003) 56(9):833–42. 10.1016/S0895-4356(03)00155-0 14505767

[111] Rogosa D On the relationship between the Johnson-Neyman region of significance and statistical tests of parallel within-group regressions. Educ Psychol Meas (1981) 41(1):73–84. 10.1177/001316448104100108

[112] Bauer DJ Curran PJ Probing interactions in fixed and multilevel regression: inferential and graphical techniques. Multivar Behav Res (2005) 40(3):373–400. 10.1207/s15327906mbr4003_5 26794689

[113] Briggs AH Claxton K Sculpher MJ Decision modelling for health economic evaluation. Oxford: Oxford University Press (2006), ISBN: 9780198526629.

[114] McGorry P Prevention, innovation and implementation science in mental health: the next wave of reform. Br J Psychiatry (2013) 202(s54):s3–s4. 10.1192/bjp.bp.112.119222 23288498

[115] Cuijpers P van Straten A Warmerdam L van Rooy MJ Recruiting participants for interventions to prevent the onset of depressive disorders: possible ways to increase participation rates. BMC Health Serv Res (2010) 10(1):181. 10.1186/1472-6963-10-181 20579332 PMC2907376

[116] Griffiths KM Christensen H Jorm AF Predictors of depression stigma. BMC Psychiatry (2008) 8(1):25. 10.1186/1471-244X-8-25 18423003 PMC2386456

[117] Davies EB Morriss R Glazebrook C Computer-delivered and web-based interventions to improve depression, anxiety, and psychological well-being of university students: a systematic review and meta-analysis. J Med Internet Res (2014) 16(5):e130–e130. 10.2196/jmir.3142 24836465 PMC4051748

[118] Mohr DC Spring B Freedland KE Beckner V Arean P Hollon SD The selection and design of control conditions for randomized controlled trials of psychological interventions. Psychother Psychosom (2009) 78(5):275–84. 10.1159/000228248 19602916

[119] Mohr DC Ho J Hart TL Baron KG Berendsen M Beckner V Control condition design and implementation features in controlled trials: a meta-analysis of trials evaluating psychotherapy for depression. Transl Behav Med (2014) 4(4):407–23. 10.1007/s13142-014-0262-3 PMC4286544 25584090

[120] Cuijpers P Cristea IA What if a placebo effect explained all the activity of depression treatments? World Psychiatry (2015) 14(3):310–1. 10.1002/wps.20249 PMC4592653 26407786

[121] Cuijpers P Meta-analyses in mental health research: a practical guide. Amsterdam: Vrije Universiteit Amsterdam (2016), ISBN: 9789082530506

[122] Lieber RL Statistical significance and statistical power in hypothesis testing. J Orthop Res (1990) 8(2):304–9. 10.1002/jor.1100080221 2303964

[123] Field A Discovering statistics using IBM SPSS statistics. London: sage (2013), ISBN: 1446274586

[124] Farrer L Gulliver A Chan JK Batterham PJ Reynolds J Calear A Technology-based interventions for mental health in tertiary students: systematic review. J Med Internet Res (2013) 15(5):e101. 10.2196/jmir.2639 23711740 PMC3668609

[125] Davies EB Morriss R Glazebrook C Computer-delivered and web-based interventions to improve depression, anxiety, and psychological well-being of university students: a systematic review and meta-analysis. J Med Internet Res (2014) 16(5):e130. 10.2196/jmir.3142 24836465 PMC4051748

